# Risk Factors of Malignant Glaucoma Occurrence after Glaucoma Surgery

**DOI:** 10.1155/2017/9616738

**Published:** 2017-08-24

**Authors:** Karolina Krix-Jachym, Tomasz Żarnowski, Marek Rękas

**Affiliations:** ^1^Department of Ophthalmology, Military Institute of Medicine, Szaserów Street 128, 04-141 Warsaw, Poland; ^2^Department of Diagnostics and Microsurgery of Glaucoma, Medical University of Lublin, Chmielna Street 1, 20-079 Lublin, Poland

## Abstract

**Purpose:**

The aim of this study was twofold: first, to evaluate the predisposing factors for occurrence of malignant glaucoma and second, to compare frequency of malignant glaucoma depending on the type of primary glaucoma surgery.

**Methods:**

Retrospective analysis was performed in 1689 consecutive patients who underwent glaucoma surgery alone or combined with phacoemulsification. Data collected included the type of surgery, width of the filtration angle, presence or absence of malignant glaucoma in the postoperative period, and time from the primary surgery to malignant glaucoma occurrence.

**Results:**

Malignant glaucoma occurred in 22 eyes that amounted to 1.3% of cases among all surgery performed. Mean time from glaucoma surgery to malignant glaucoma occurrence was 61.4 ± 190.5 days. Among patients with penetrating surgery, malignant glaucoma occurred in 2.3% of patients, whereas after nonpenetrating operations, such complication was not found (*p* = 0.00004). Malignant glaucoma occurred more often in patients with shallow iridocorneal angle (*p* = 0.0013).

**Conclusions:**

The risk of malignant glaucoma development is associated with penetrating characteristic of glaucoma surgery, after which this complication appears and its occurrence is higher in eyes with shallow iridocorneal angle. The risk of malignant glaucoma after trabeculectomy compared to iridencleisis as well as after phacotrabeculectomy compared to phacoiridencleisis is equivalent.

## 1. Introduction

The problem of malignant glaucoma as a complication of glaucoma surgery appears to be rare enough that large randomized trials have not been conducted yet in order to determine the relationship between its occurrence and various types of treatments or to determine treatment strategy giving the best results in large populations of patients. At the same time, it is one of the most serious complications of intraocular surgery, which in the natural course results in, often irreversible, loss of vision in a short period of time.

So far, predisposition for malignant glaucoma was found in eyes with chronic angle closure glaucoma; nevertheless, cases in which open angle glaucoma was diagnosed prior to the surgery have also been reported [1, 2]. Malignant glaucoma is more frequent in small eyes, and anatomical abnormalities in the anterior chamber are also predisposing [2, 3]. Its relationship to conditions such as hyperopia and microphthalmos (*nanophthalmos*) has been described [4–6]. It has also been determined that higher incidence of malignant glaucoma among women occurs presumably due to smaller dimensions of the anterior segment of the eye [3].

Symptoms of inflammation in the anterior segment of the eye and previous surgical procedures were also mentioned as predisposing to malignant glaucoma [1]. The high intraocular pressure prior to the surgery was suggested as a predisposing factor, but further observations have not confirmed it [7].

The aim of this study was to identify preoperative risk factors for malignant glaucoma and to assess its occurrence depending on the type and nature of the primarily conducted glaucoma surgery. Defining factors predisposing to malignant glaucoma is important due to the possibility of early diagnosis in the endangered eyes, which ultimately determines the final effect of the treatment. This is particularly important because persistent symptoms of malignant glaucoma lead to corneal oedema and its decompensation, severe anterior adhesions, cataract formation in phakic eyes, and structural and functional damage of the optic nerve associated with glaucomatous process. Unfortunately, there are no reliable methods to prevent complications such as malignant glaucoma in the predisposed eyes so far. As long as the causative factors and the aetiology of the process are not well understood, the treatment is an even greater problem.

## 2. Methods

This was a single-centre, retrospective study of glaucoma patients, and the study protocol was approved by the Institutional Review Board. Retrospective analysis was performed in 1689 consecutive patients (1061 women and 628 men) who underwent glaucoma surgery or combined cataract and glaucoma surgical treatment.

Among the procedures performed in the study group, there were methods of surgery: trabeculectomy, nonpenetrating deep sclerectomy, iridencleisis, Ahmed valve implantation, Gold Micro Shunt (GMS) implantation, Ex-Press implantation, and a group of “other” treatments that have been performed rarely and therefore analysed jointly: implantation of i-Stent, viscocanalostomy, CyPass implantation, and canaloplasty.

Iridencleisis is a quite old and rarely performed free-filtering procedure that creates a full-thickness fistula between the anterior chamber and the subconjunctival space through an anterior sclerostomy. The procedure involves entrapping a pillar of iris tissue into the sclerostomy to act as a wick to hold the sclerostomy open. From own experience, it is an efficacious surgical approach in patients with shallow iridocorneal angle when it comes to intraocular pressure (IOP) reduction. According to our observation, this type of surgery has favorable safety profile than trabeculectomy especially in eyes which are surgically challenging, in nanophthalmos and in eyes with crowded structures in the anterior chamber.

The decision to perform a combined procedure depended on vision loss connected with cataract development, the number of antiglaucoma medications used, and the stage of glaucoma. Subtype of glaucoma surgery was chosen individually for each patient.

Glaucoma surgery was classified as follows:

- Depending on the combination of glaucoma procedure with phacoemulsification:
  - Glaucoma surgery without phacoemulsification
  - Glaucoma surgery combined with phacoemulsification
- Depending on the type of the aqueous humour main drainage pathway:
  - Penetrating surgery (trabeculectomy, iridencleisis, seton implantation, surgery with implantation of Ex-Press, and treatments mentioned above combined with phacoemulsification)
  - Nonpenetrating surgery (nonpenetrating deep sclerectomy, i-Stent, CyPass and GMS implantation, viscocanalostomy, and canaloplasty both as a sole surgery and combined with phacoemulsification).

Collected data include the date and type of surgery, age and sex of the patient, operated eye, width of the chamber angle, the power of the implanted IOL for procedures combined with cataract surgery, and presence or absence of malignant glaucoma in the postoperative period. Consecutive patients who, after the primary surgery, experienced symptoms of malignant glaucoma, defined as a progressive increase in IOP associated with the axial shallowing of the anterior chamber in the presence of a patent iridotomy, were included into the analysed group. Medical history was collected regarding previous surgical and laser procedures; additional examinations included IOP, BCVA, anterior segment with anterior chamber depth assessment, ocular fundus with the evaluation of c/d, the measurements of CCT and AXL, ultrasound B-scan, and OCT examination of the anterior segment of the eye, if necessary. Iridocorneal angle was classified as shallow if it measures 0–20° (grade 0–2 according to Shaffer-Etienne Classification System) and wide if it measures 20–45° (grade 3-4 according to Shaffer-Etienne Classification System).

### 2.1. Statistical Analysis

Statistical analysis of the investigated variables was performed with the Shapiro-Wilk and paired Wilcoxon tests. Friedman ANOVA for matched groups and rank means and rank sums were also used for post hoc comparison. The Kaplan-Meier method was used to determine survival curves, and differences between them were tested by the log-rank test. A *p* value of 0.05 or less was considered significant. The calculations were performed with Statistica 10.0 PL.

## 3. Results

### 3.1. Demographic Data

The studied material included 1689 eyes treated with glaucoma surgery in a single clinic (Military Institute of Medicine in Warsaw), where 1061 accounted for women's eyes (62.8%) and 628 for men's eyes (37.2%). In the study group, 811 of the eyes were right, which accounted for 48.0% of the material, and the left eyes counted 878, which accounted for 52.0%. The number of 960 (56.8%) penetrating operations and 729 (43.2%) nonpenetrating operations have been conducted. The number of 1417 (83.9%) operations were combined with phacoemulsification, while glaucoma surgery without cataract surgery amounted to 272 cases (16.1%).

The filtration angle was wide opened in 1210 cases (71.6%), while it was shallow in 479 eyes (28.4%). Mean patients' age was 72.9 ± 10.6 years (Me 75) within the range of 16 to 95 years. Women's mean age was 73.2 ± 10.2 years (Me 75) within the range of 20 to 93 years. Men's mean age was 72.4 ± 11.3 years (Me 75) within the range of 16 to 95 years. The mean time elapsed from primary glaucoma surgery was 61.4 ± 190.5 days (Me 2.5; from 1 to 840 days). In the analysed material, malignant glaucoma occurred in 22 eyes, which accounted for 1.3% of the whole group (Table 1).

Analysis of the types of glaucoma surgery in whole group of patients is presented in Table 2.

In the group of patients with malignant glaucoma, 40.9% of eyes underwent surgical treatment with the method of phacoiridencleisis, 22.7% phacotrabeculectomy, 18.2% iridencleisis, 13.6% trabeculectomy, and 4.5% seton valve implantation before this complication occurred.

### 3.2. Analysis of the Types of Glaucoma Surgery

#### 3.2.1. Analysis of Malignant Glaucoma Occurrence Depending on the Type of Glaucoma Surgery

Among patients with penetrating surgery, malignant glaucoma occurred in 2.3% of patients, whereas after nonpenetrating surgery, this complication was not found (*p* = 0.00004) (Table 3). The conclusion is that penetrating surgery is the risk factor of malignant glaucoma occurrence.

#### 3.2.2. Analysis of Malignant Glaucoma Occurrence Depending on the Subtype of Glaucoma Surgery

The risk of malignant glaucoma after phacotrabeculectomy and phacoiridencleisis was equivalent (*p* = 0.810). When frequency of malignant glaucoma after trabeculectomy and iridencleisis was compared, the difference was not statistically significant (*p* = 0.416) (Table 3).

#### 3.2.3. Analysis of Malignant Glaucoma Occurrence Depending on Sex of the Patients

Malignant glaucoma was more frequent in women, but the difference was not statistically significant (*p* = 0.064) (Table 3).

#### 3.2.4. Analysis of Malignant Glaucoma Occurrence Depending on Iridocorneal Angle Width

This complication occurred more often in patients with shallow iridocorneal angle (*p* = 0.001). The risk of malignant glaucoma is 3 times higher in eyes with shallow filtration angle (Table 3).

## 4. Discussion

Understanding of malignant glaucoma since it was first reported in 1869 has extended, but still many questions remain unanswered.

The diagnostics of malignant glaucoma mechanisms enables using of specific treatment aimed at pathophysiological process being the underlying cause, but in fact different mechanisms may coexist, consequently complicating the diagnosis [8]. The theory of malignant glaucoma pathogenesis involves reversed outflow of fluid towards or beyond the vitreous body, with subsequent increase in volume of the vitreous and shallowing of the posterior and anterior chamber [9].

In the initiation of this complication, both high lens: eye index (lens typically has a normal or increased thickness) [5] and abnormal histological structure of the sclera may play a role. A disproportionately large lens in relation to the anterior segment of the eye may indicate higher risk of malignant glaucoma incidence [10]; the relation between the size of the lens and the scleral ring or the top of the ciliary body is critical [1]. Disrupted structure of collagen fibres of the intercellular substance of the sclera's connective tissue [11] and increased levels of fibronectin have been found, as well as changes in the metabolism of glycosaminoglycans, which can cause compression of the collagen fibres and lead to thickening of the sclera. Thickening of the sclera may, on the other hand, cause partial narrowing of the vortex veins, impairing normal venous drainage [5], while its smaller surface decreases the transscleral protein transport. As a consequence of these predisposing factors, the choroidal bed becomes overflown (CE—*choroidal effusion*) [5, 12]. All of these features and increased oncotic pressure of the vitreous body may be associated with a higher risk of developing malignant glaucoma [5, 12]. It is important to recognize anatomical predisposing factors and pathophysiological changes leading to this process as a mechanism for introducing full-blown malignant glaucoma because the treatment of this complication is challenging.

In this group of patients when conservative management (mannitol intravenously, acetazolamide p.o., and locally: 1% atropine, 1% tropicamide, dorzolamide hydrochloride-timolol maleate ophthalmic solution, and 0.1% dexamethasone phosphate) was insufficient, laser treatment was recommended. Capsulotomy was performed using an energy of 1 to 4 mJ per pulse. The energy and pulses were modified according to the thickness of the capsule until an opening was achieved. 5–15 bursts with an energy of 1–3 mJ through iridotomy or iridectomy were usually effective in achieving communication, although some cases were refractory and symptoms of malignant glaucoma reappeared. The indication for surgical intervention was a lack of effectiveness of conservative and laser treatment. Partial PPV with peripheral lens capsule excision communicating anterior chamber and vitreous cavity was performed with satisfying results [13].

The occurrence of malignant glaucoma is most often a consequence of glaucoma surgery, although it was also reported in patients after laser and other ophthalmic procedures, even in the surgically nontreated eyes. In the analysed material encompassing eyes after glaucoma surgery, malignant glaucoma occurred in 22 eyes, which accounted for 1.3% of the whole group. Among the penetrating operations, prevalence of this complication was 2.3% in operated eyes, which is comparable with the observations other authors have made [7, 14]. In the analysed material, malignant glaucoma was not observed after nonpenetrating surgery.

Possibly, leaving TDM (trabeculo-Descemet's membrane) intact avoids sudden decompression associated with the opening of the anterior chamber, which causes its flattening and anterior displacement of the iridolenticular diaphragm. Such mechanism is regarded by some authors as the main intraoperative cause initiating malignant glaucoma development [15–17]. The movement of the diaphragm may occur as a result of leakage of filtering bleb, which initiates a vicious cycle mechanism leading ultimately to the development of malignant glaucoma [18]. Filtering bleb leakage may be associated with excessive filtration [19]. The problem that often occurs after full-thickness surgery and, in addition, the widespread use of antimetabolites significantly increases the incidence of this complication [20]. It is believed that the aqueous humour has lytic properties and inhibits the subconjunctival fibroblasts [21, 22], leading to persistent leakage of the filtering bleb [20]. In the controlled filtration surgery, an incomplete-thickness scleral flap may also become thin over time due to the lytic properties of aqueous humour and resemble effects of full-thickness filtration surgery, promoting the development of late bleb leakage. This process is enhanced by adjuvant antimetabolite therapy [20]. However, after nonpenetrating surgery, hypotension typically occurs without changing the anatomical relations, which may limit the incidence of malignant glaucoma. Karlen et al. described a case of malignant glaucoma occurring after NPDS (*nonpenetrating deep sclerectomy*) [23]. Thus, it is difficult to completely exclude the possibility of malignant glaucoma development after NPDS, especially since in many cases the microdamages of TDM happen, which facilitates aqueous humour outflow and causes it to perform similarly as in the classic penetrating surgery [24].

The study illustrates the incidence of malignant glaucoma in particular types of surgery. Among the procedures, where malignant glaucoma occurred as a complication, were trabeculectomy, iridencleisis, seton valve implantation, and surgery combined with phacoemulsification: phacoiridencleisis and phacotrabeculectomy. Penetrating nature of the operations allows us to formulate a general trend for the occurrence of this type of complication in this particular group of surgery. After iridencleisis, malignant glaucoma occurred in 8.9% of patients, which is highly interesting, as well as the fact that after trabeculectomy, this complication was observed in 4.9% of patients. The probable cause of this result was the fact that iridencleisis was performed in cases of narrow iridocorneal angle glaucoma as the operation of choice and predilection for malignant glaucoma are associated with this configuration [25].

When glaucoma coexisted with cataract, glaucoma operations were carried out combined with phacoemulsification. Phacotrabeculectomy was performed mainly in eyes with open iridocorneal angle glaucoma, while in the hyperopic eyes, microphthalmos, or relative anterior microphthalmos, the preferred surgical technique was iridencleisis combined with cataract surgery because of lower tendency of postoperative shallowing of the anterior chamber after mentioned procedure. In the analysed material, malignant glaucoma occurred in 1.81% of patients after phacotrabeculectomy, while after phacoiridencleisis, this complication was observed in 2.07% of patients. No differences were found in the incidence of malignant glaucoma, but it should be noted that the phacoemulsification with iridencleisis in the predisposed eyes could stabilize the anatomical relationships in the anterior segment of the eye.

The time of onset of malignant glaucoma symptoms from the primary glaucoma surgery differed significantly in the study group. Malignant glaucoma may have different dynamics of clinical manifestation immediately after surgery, when the causative factors cannot be compensated in a closed system of the eyeball. On the other hand, symptoms may be delayed if a relative balance between the volume of produced fluids and the drainage from the eyeball is established.

It appears that disturbed anatomical relations between the anterior and posterior segment of the eyeball underlie the pathogenesis of malignant glaucoma. According to some authors, eyes with malignant glaucoma tend to coexist with hyperopia with AXL < 22 mm [4]. Average AXL in patients with malignant glaucoma in our study was 21.79 ± 0.83. In the papers by Byrnes et al. (21 eyes) and Żarnowski et al. (10 eyes), the average AXL in malignant glaucoma patients was 21.15 mm and 21.30 mm, respectively [26, 27]. Among the examined patients, average anterior chamber depth was 2.0 ± 0.8 mm preoperatively, while in the normal adult population, the average chamber depth is 3.15 mm [28].

Taken together, assessment of factors that predispose to malignant glaucoma and knowledge of its occurrence depending on the type and nature of the primarily conducted glaucoma surgery might draw attention to the eyes at risk of developing this complication.

Filtration surgery in the predisposed eyes can be modified to reduce the incidence of this complication, where the aim is to keep the iridolenticular diaphragm in the anatomically correct position, which seems more probable when nonpenetrating procedures are used, although they are not always possible due to the anatomical relation occurring prior to the surgery. However, excessive filtration should be avoided as it may cause anterior chamber flattening and initiate the malignant process. Any inflammatory processes and a history of repeated surgical interventions in the eye prepared for the procedure should be taken into careful consideration, as well as a history of malignant glaucoma in the fellow eye.

## Figures and Tables

**Table 1:** Patient's demographic data.

Demographic data	Mean ± SD (Me)
*Sex n* (%)	
Women	1061 (62.8%)
Men	628 (37.2%)
Together	1689 (100.0%)

*Eye n* (%)	
Right	811 (48.0%)
Left	878 (52.0%)

*Surgery n* (%)	
Penetrating	960 (56.8%)
Nonpenetrating	729 (43.2%)

*Surgery n* (%)	
Combined with phacoemulsification	1417 (83.9%)
Glaucoma surgery without phaco	272 (16.1%)

*Iridocorneal angle n* (%)	
Wide	1210 (71.6%)
Shallow	479 (28.4%)

*Malignant glaucoma occurrence n* (%)	
Malignant glaucoma	22 (1.3%)
Without malignant glaucoma	1667 (98.7%)

*Age (years)*	72.9 ± 10.6
	Me 75 (69.80)
Range	16.0–95.0

**Table 2:** Percentage distribution of surgical methods in analysed material.

	Surgery	Number of surgeries (*n*)	%
1	Phacosclerectomy	545	32.3
2	Phacoiridencleisis	435	25.8
3	Phacotrabeculectomy	276	16.3
4	Deep sclerectomy	110	6.5
5	Phaco-ExPress	105	6.2
6	Trabeculectomy	61	3.6
7	Iridencleisis	45	2.7
8	Phaco-GMS	43	2.5
9	Seton valve implantation	21	1.2
10	Other	17	1.0
11	GMS	14	0.8
12	ExPress	14	0.8
13	Phaco-seton valve implantation	3	0.2
Together		1689	100.0

**Table 3:** Analysis of malignant glaucoma occurrence depending on different factors.

Analysed data		Malignant glaucoma			
		Yes	No	Together	*p* (Chi^2^)
Glaucoma surgery	Penetrating	22	938	960	0.00004
	Nonpenetrating	—	729	729	
	Together	22	1667	1689	

Subtype of glaucoma surgery	Phacotrabeculectomy	5	271	276	0.810
	Phacoiridencleisis	9	426	435	
	Together	14	697	711	
	Iridencleisis	4	41	45	0.416
	Trabeculectomy	3	58	61	
	Together	7	99	106	

Sex	Women	18	1041	1059	0.063
	Men	4	622	626	
	Together	22	1663	1685	

Iridocorneal angle	Wide	9	1201	1210	0.001
	Shallow	13	466	479	
	Together	22	1667	1689	

## Additional Points

*Limitations of the Study*. There are some limitations of the current study. First, retrospective data commonly has more sources of error due to confounding and bias. Second, the sample size was too small to detect slight differences between groups. It is difficult to collect a large number of cases since malignant glaucoma is a very rare disease. Prospective trials with greater statistical power will be needed to detect more detailed predisposing factors for malignant glaucoma. *Summary*. The risk of malignant glaucoma development is associated with eye anatomical relations and is particularly high in the eyes with shallow iridocorneal angle. Nonetheless, the complication does not occur in all cases of the eyes with anatomical predispositions. The cause may lie in the histological structure and existing biochemical features, which are not yet discovered. Malignant glaucoma occurrence is also linked to penetrating characteristic of glaucoma surgery, after which this complication appears. The risk of malignant glaucoma after trabeculectomy compared to iridencleisis as well as after phacotrabeculectomy compared to phacoiridencleisis is equivalent.
